# The death of a patient: a model for reflection in GP training

**DOI:** 10.1186/1471-2296-12-8

**Published:** 2011-03-03

**Authors:** Anita AH Verhoeven, Jan Schuling, Els LM Maeckelberghe

**Affiliations:** 1 Department of General Practice, University Medical Center Groningen, University of Groningen, PO 196, 9700 AD Groningen, the Netherlands; 2 Expertise Centre Ethics of Care, University Medical Center Groningen, the Netherlands

## Abstract

**Background:**

The Dutch government has chosen a policy of strengthening palliative care in order to enable patients to die at home according to their preference. In order to facilitate this care by GPs, we wanted to know how to support them in their training. Therefore we examined the ways in which the death of a patient influences the doctor both at a professional and at a personal level.

**Methods:**

Based on a qualitative study, we developed a model for reflection for GP trainees on the meaning of the death of patients and its influence on the GP.

The qualitative study was done in 2007 and is based on open in-depth interviews and a focus group. We recruited 18 participants who were highly professional GPs and experienced in talking about the death of patients. We invited GPs from a list of experienced GPs, who in addition are also second-opinion GPs for euthanasia (SCEN-physicians) and from a pool of GP trainers, our intention being to include GPs holding a variety of world views. Interviews were audio-taped and transcribed verbatim. A grounded theory approach was used to analyze the results. Themes were first identified independently by three researchers, then after discussion these three sets were rearranged to one list of themes and their mutual relation were determined. A model for the interaction of the GP at professional and at a personal level was formulated.

**Results:**

Forty three themes emerged from the interviews and focus group. These themes fell into three groups: professional values and experiences, personal values and experiences, and the opinions of the GPs as to what constitutes a good death. We constructed a model of the doctor-patient relationship on the basis of these findings. This model enables GP trainees identifying the unique character of the doctor-patient relationship as well as its reciprocity when the two were confronted by the patient's impending death.

**Conclusions:**

In dealing with the approaching death of a patient the unique interaction between patient and doctor and the cumulative experiences of doctors with their patients brings about a shift in the GP's own values. The professional development of GP trainees may be facilitated by reflection on the interaction of their own values and beliefs.

## Background

In the Netherlands, the GPs' role at the end of their patients' lives changes due to some recent developments. We mention three processes that are of interest. First of all, there is a demand for improvement in the quality of palliative care regarding where the patient dies: at home, in a hospital, a hospice, or elsewhere. It is an important indicator of the quality of palliative care [1]. Although 75% of people in the Netherlands would prefer to die at home, only 33% actually do so [2]. Hence the Dutch government has chosen a policy of strengthening palliative care in the home [3]. This policy is implemented by supporting general practitioners (GPs) who provide palliative care at home. Eventually, this might contribute to an increase in the number of patients who choose to die at home. We claim that implementation of this policy should be flanked by reflective support concerning GPs own understanding of dealing with death and dying.

A second development in general practice is the introduction of large scale out-of-hours services in primary care. These services have resulted in a loss of contact on the part of the GPs with their own patients during these hours and therefore in care for a patient dying at home being provided by unfamiliar GPs (locums). Such a development seems to run contrary to the emphasis on personal continuity in care for the patient who dies at home.

A third change in our western society and therefore in general practice is the increasing call for interest in spiritual issues, as they affect medicine [4,5]. The WHO has pointed to spirituality as one of the six indicators of quality of life in different cultures [6]. In palliative care especially there is an increasing emphasis on spirituality as a factor which contributes to the patient's wellbeing and provides strategies for coping with illness [7,8]. Attention to spirituality is not self-evident for Dutch doctors who work in one of the most secularized countries in the world [9].

These three processes ask for self-reflection of the doctors in caring for their patient.

In the literature we found empirical studies (table 1) [10-15] as well as models (table 2) [16-21] of the doctor-patient relationship appropriate for use in the case of a dying patient. In the empirical studies on how GPs deal with the death of a patient, we found three types of influencing factors: those related to the physician, the environment and the patient.

**Table 1:** Empirical studies

Main characteristics of empirical studies on how physicians deal with the death of a patient *			
Studies	Year, participants, country	Type of study	Goal of study; conclusion
Moores 2007	2005 188 hospital doctors UK	Questionnaire	Memorable patient deaths; Almost 50% of physicians feel sad following a memorable patient death;around 1 in 10 experiences physical and emotional responses of moderate to severe intensity. The experience of a memorable patient death was influenced by personality traits more than by medical experience

Jackson 2005	1999-2001 51 physicians of quaternary care medical centres USA	90-minutes semi structured interviews; face-to-face survey on a ten-point scale	To understand emotional experiences of physicians in caring for dying patients; Physicians' emotional reactions to patient death may affect patient care and the personal lives of physicians

Redinbaugh 2003	Before 2003 188 hospital doctors, including primary care physicians USA	Semistructured interviews, face-to-face questions and a self administered questionnaireeducation	Doctors' emotional reaction to the recent death of an 'average' patient; effect of level of training; A long and close relationship with a patient makes the doctor more vulnerable to feelings of loss; doctors may benefit from debriefing

Hoogerwerf 1999	Before 1999 25 physicians(opinion-leaders) the Netherlands	Interviews	Factors influencing medical decision-making at the end of life; For 80% of the physicians, personal values and experiences were important in medical decision making at the end of life

Saunderson 1999	1996-1998? 25 GPs UK	Semistructured interviews	Managing one's own bereavement and that of the patient; GPs may need support and learning methods to manage their own bereavement

Durand 1990	Before 1990 441 family physicians USA	Two-page fixed-choice questionnaire	Personal attitude toward death; feelings and reactions toward terminally-ill patients and their families; A significant relation between having a positive attitude to death and being over 50, having a religious world view and having received education concerning death

*Resuls of literature search in PubMed, Embase, PsycInfo, Cinahl, TRIP database, Web of Science, Scholar.google.nl and the University of Groningen catalogue (publication date 1980 - 2008).

Search terms used for physicians and general practice: physician(s), doctor(s), family physician(s), family practice, general practitioner(s), GP(s), general practice

for death: death, attitude to death, suffering, grief, bereavement, end of life, and patient loss.

**Table 2:** Models on doctor-patient relation suitable for caring for dying patients

Model	Context	Components
Leget 2007	Spiritual care in palliative medicine; philosophical view	Five themes of dying well approached from an inner space:
		1. autonomy and the self
		2. pain control and medical intervention
		3. attachment and relations
		4. life balance and guilt
		5. death and afterlife

Meier 2001	Palliative care	Psychological model of doctor's emotions that may affect patient care;
		Description of high risk situations. Goal is self-awareness

Redinbaugh 2001	Palliative care Viewpoint of occupational medicine	Model for health care professionals coping with grief reactions

Emanuel and Emanuel 1992	Physician-patient relation in general	Four models of physician-patient relation characterized by four variables (goal of physician-patient interaction, physician's obligations, role of patient's values, patient autonomy):
		1. paternalistic model
		2. informative model
		3. interpretive model
		4. deliberative model

Steinmetz 1992	Family physician's role in care for the dying; aimed at research and education	Three dimensions:
		- care for patient and family
		- self care
		- cooperation with other health care professionals
		Each dimension has three levels in relation to complexity and intimacy of relations.

Spreeuwenberg 1981	General practice; care for the dying	Norms, values, beliefs of GP as well as patients; fundamental attitude of GPs to dying patients

### The physician

- Emotions: guilt and blame, fear of making mistakes, anger, feelings of helplessness, emotional closure, isolation, feeling upset when thinking about the patient, feeling numb, sadness, relief

- Physical reactions: hyperactivity or hypo-activity, changes in appetite, tiredness, sleeping problems

- Personal experiences of loss (within family or inner circle)

- Personal style of coping and of the management of one's own bereavement and that of the patient

- Identification with patients and their families

- Questions of personal competency

- Intellectual closure

- Sense of (moral) responsibility for the death

- Perspective on dying: physic-pathology, psycho-social perspective, meaning and purpose of life

- Metaphors of death:

• Death as the absolute end (e.g. burned down candle)

• Death as a way of continued life (e.g. resurrection)

• Death as a process which belongs to life

### The environment

- Cultural and religious context

- Contact with bereaved patients

- Professional experiences

- Guidance from colleagues

- Education on death

### The patient

- Length of patient-physician relationship

- Intensity of patient-physician relationship

- Type of death (shocking, unexpected, 'good', 'over-treated')

In Moores' study, only those topics are mentioned which were rated on a 4-point scale as moderate (3) or severe (4) and were scored by more than 10% of the participants.

In the present study, we examine how GPs deal with a dying patient and with their death. Our goal was to create a model to be used in the training of GPs. This model enables them to reflect on how diverse elements contribute to their own conception of what a good death might be for a patient and themselves.

## Methods

### Study design

In order to formulate our reflection model, we performed qualitative research.

We conducted interviews and a focus group (or group interview). These qualitative methods were used because there is little empirical knowledge about this topic. Individual interviews were chosen because they increase the privacy of the participants and hence their ease and comfort in disclosing their feelings. Since we were interested in the inner life of GPs and in what the experience of a patient's death meant for them, the use of interviews provided flexibility for discussing an issue more deeply when appropriate. As a supplementary method, we chose a focus group because we expected that interaction between participants would stimulate discussion. However, we found that individual interviews revealed higher quality statements than group interviews. So after conducting five individual interviews and one focus group, we continued with individual interviews only. After a total of nine individual interviews we stopped, for data saturation had been achieved. In qualitative research the aim is not to select at random a representative number of participants. The aim is to collect as many views as possible either coming from a small number or a larger number of participants. It does not matter when many statements come from one GP. The statements matter, not who made the statements.

The interviews were held between May and December 2007. The individual interviews lasted one hour on average, and the group interview two hours. After the interview the GPs received a book token of €30 for their participation in the study.

### Recruiting GPs for interviewing

After a pilot interview with one of the GPs of the Academic Primary Health Care Center of our university to explore the topic, we started to recruit GPs for interviewing.

To obtain a diversity of opinions and experiences we invited GPs with a variety of religious and secular world views and coming from practices with different characteristics. At our request the Royal Dutch Medical Association, which runs the registration of SCEN doctors, invited all the SCEN-doctors from the north of the Netherlands by letter (SCEN-doctors: Support and Consultation on Euthanasia in the Netherlands [22]). SCEN-doctors are experienced GPs, who, in addition, are also second opinion doctors for euthanasia for their colleagues. A SCEN-doctor from the north of the Netherlands performs six second-opinion consultations on average a year (range 0 - 12) and gives advice concerning palliative care twice [23]. Typically, one consultation takes four hours including contacting the requesting physician, studying the records, visiting the patient and writing the report. These GPs have a lot of experience with dying patients and are used to communicating and to reflecting on death and dying and were therefore suitable for the purposes of our study.

In the Netherlands, euthanasia is understood to mean the termination of life by a doctor at the patient's request, with the aim of putting an end to unbearable suffering with no prospect of improvement. It includes suicide with the assistance of a doctor. The voluntary nature of the patient's request is crucial: euthanasia may only take place at the explicit request of the patient. This Dutch definition of euthanasia contrasts with the definitions used in some countries, where it is sometimes interpreted as meaning termination of life by a doctor without the consent of the patient [24].

Of the 70 GPs who were invited, 21 agreed to be interviewed. From this group four GPs were randomly chosen to be interviewed. Because more of the SCEN doctors than we had expected agreed to participate, we invited the other 17 GPs by letter to participate in a group interview. After arranging a date, nine of these GPs were actually able to participate in the group interview.

In order to broaden the individual interviews we included an additional four GPs from the pool of GP trainers cooperating with the department of vocational training for general practice. They were selected to complement the other participants because of their specific characteristics which were only partially represented among the first four. These were their age (under 50), sex (female), world view (anthroposophy or Christian) or the characteristics of their practices (large ethnic minority patient groups, practice location).

The authors have complied with 1. The Medical Research Involving Human Subjects Act http://www.ccmo.nl and 2. the UMCG-research code, the research code of the University Medical Centre of Groningen where the authors work http://www.rug.nl/umcg/onderzoek/researchcode/index. No formal ethics approval is required according to these national and local guidelines. The research, however, was done following ethical research standards. Special attention was given to confidentiality and privacy. All participants received their anonymously-made interview to check and all agreed with it. They were aware the study would be published with their direct quotes. After finishing the study, they received an electronic version of the Master's thesis by mail and a link to thesis on the website of the author http://www.rug.nl/staff/a.a.h.verhoeven/research?lang=en.

### Questionnaire

Before the start of the individual and group interviews the GPs filled in a brief questionnaire with nine questions about themselves and their practice such as age, years of experience and world view [Additional file 1].

### Interviews, interview question and interview technique

We started with a pilot interview using a detailed interview scheme. In the evaluation this turned out to be too restrictive as it seemed to obstruct the GP's expression of his recollection of the situation. We therefore changed the interview scheme of the individual interviews by asking just one question about memorable patient deaths: "Can you tell me about a case of a patient who died, maybe whose death lingers on in your memory?" This question opened a plethora of experiences. No new topics were raised by the interviewer subsequently; only topics mentioned by the interviewee were discussed more thoroughly.

The principal investigator (AAHV) was also the interviewer for all the individual interviews. She was trained as a GP, worked at the Department of General Practice and was engaged in this present study as the completion of her Masters in Spiritual Counseling.

The group interview was led by an external moderator who was an experienced group facilitator for GPs.

### Data registration and data analysis

The qualitative method used was based on a grounded theory approach to data analysis in which theories are generated from the data [25]. The qualitative method used was inspired by the grounded theory using the constant comparative method.

The interviews were audio-taped and transcribed verbatim immediately after each interview. The interviewer checked each transcription against the tapes and then read them for accuracy. Each interview was then sent to the interviewee for their comments (member check). Only minor changes were suggested. Anonymity and confidentiality were guaranteed by changing the names of persons, places, jobs and characteristics of the patient or the doctor when these were unique.

Qualitative analysis was conducted on the transcripts of the interviews by three investigators, the authors of this study. All of them have different backgrounds: the principal investigator is a trained GP and a student in Spiritual Counseling, the second analyst is a theologian and ethicist working in a hospital setting (ELMM), and the third is an experienced GP and senior researcher in general practice (JS). Analysis was guided by the grounded theory, which seeks to develop and understand connections between main themes and among them. The interviews produced a multitude of interesting topics. In the analysis, the investigators aimed at clarifying the material in such a way that it could be translated into a functional, practical and theoretically insightful model for GP trainees.

The three investigators independently identified key words and phrases and coded them with self-formulated themes. They discussed the results of the coding in order to identify inconsistencies and to determine the relevance of their interpretations. During the coding process the interview texts related to the themes were read and reread for accurate interpretation. Disagreements were resolved by persuasive arguments using the interview text. When eventually a list of themes emerged and was agreed upon, three main themes were formed. Finally, the relationship between these categories was determined and expressed in a doctor-patient model.

## Results

### The doctor-patient model

After analysis and discussion of the 160 pages of interviews, 43 main themes were identified, associated with the memorable death of a patient, as recalled by a GP (table 3). Thirteen themes had a high internal consistency, intensity of the words spoken and specificity of the experience ("I ..." statements). Within the 43 themes we identified three uniting themes: professional values and experiences, personal values and experiences, and statements about what a good death or a good process of dying may be.

**Table 3:** 43 themes emerging out of interviews with 18 GPs about the death of a patient

**A.Good death (or not)**	**B.Professional values and experiences**	**C.Personal values and experiences**
1. Good farewell*	1. Being a backstage director*	1. Feelings of guilt, responsibility and powerlessness*

2. Holding on and letting go by the patient; balance in autonomy*	2. Nearness and distance; the doctor as a person in the relationship*	2. Purpose and meaning of life, death and suffering*

3. Orientation of patient towards expressing and sharing feelings and thoughts*	3. Guidance (not steering); being available for patients*	3. View on suffering: how much is a patient allowed to suffer, to what extent does suffering belong to life*

4. Peace	4. Typical tasks of the GP*	4. (Hidden) mission*

5. Beautiful	5. Helpful	5. Closure of life*

6. Effect of stage of life; degree of prematurity	6. Giving room and paying attention	6. Personal experiences with death; relation with one's own death*

7. Fits into a person's life	7. Professional responsibility	7. Religion

8. Availability and accessibility of the GP	8. GP as privileged partner with privileged knowledge	8. Partner in intimacy; admiration and amazement

9. Nature and intensity of disease	9. Showing and experiencing respect	9. Leaving one's own norms behind

10. Environment	10. Carefulness	10. Grief

11. Religion or world view	11. Self-care	11. Internalization of norms

12. Feeling that one is part of a larger entity	12. Doctor as a professional in the relationship	12. Sharing with colleagues or at home as a way of dealing with experiences

	13. Medicine as an art	13. Experiences in life and in the profession

	14. Euthanasia	14. Identification with patients

		15. Influence of doctor-patient relationship

		16. Pleasure in recognizing and identifying the patient as a person

		17. Learning from the death of others; absorbing experiences

* Themes with highest internal consistency, intensity of the words spoken and specificity of the experience ("I ..." statements).

Based on this analysis of the material provided by the interviews and the focus group we constructed a model that encompasses the variety of experiences the doctors advanced and that is didactically adequate and easily applicable in training sessions.

The model expresses the relationship between three main themes in a doctor-patient model [Additional file 2].

These three groups of statements made by the GPs are influenced by the doctor-patient relationship and embedded in it. There is a mutual influence between the doctor and the patient as for instance, in the case where the doctor agrees with a patient's request for euthanasia although he is against it. This is indicated by arrows pointing both ways. The patient to whom the arrows point is a unique patient. The doctor and patient may together make a final decision concerning the patient's care which would be different in the case of another patient. The doctor-patient relationship on its part is influenced by society and culture, for example, the acceptance of the out-of-hours service and medical technology at home. This is shown in the model by the square that surrounds it.

We will now exemplify how the three main themes organize the experiences of the GPs. We will use representative quotations from the interviews, followed by the number of the interviewee and the page number of the interview text where the quotation can be found. For the list of interviewees see [Additional file 3].

### Professional values and experiences

One family resemblance of themes was that of professional values and experiences. The GPs talked about (1) the doctor-patient relationship, (2) the professional GP and death, (3) the responsibility of the GP, (4) the privileged position of the GP, and (5) the influence of society on GPs' care for dying patients.

(1) In the interviews on the death of their patients, a recurring topic was the GPs' view on an ideal relationship with a patient. They advocated an open relationship in which their desire was to support and guide the patient, without taking the lead.

"My part in this, and I chose it to be, is to be there for people, to have an open relationship with them and to have them feel they can come to see me with anything, no matter how crazy." (GP 7, p.5)

"That you can give space to people. It's their process, their path, but you can be there to guide and support them." (GP 7, p.7)

Another description of the GP's role was that of a backstage director: somebody who carefully organises and supports. This care includes a calm oversight of the period before the moment of dying.

"It makes you sort of the director of the process. You try to direct the communication somewhat, so that people stay connected to how they feel." (GP 7, p.6)

The GPs appreciated mutual respect in the relationship with the patient with recognition and acknowledgment of each other as persons. The GPs stressed the danger of a doctor-patient relationship being either too close or too superficial. Such a relationship may hinder the communication.

(2) Many GPs thought that caring for dying patients was a primary task for GPs: it belongs to the essence of their profession.

"It may sound a bit morbid, but when I learn that one of my patients will die, I think to myself: ah, at least there is someone dying again, now I can get busy. I call it the real bonus in my work." (GP 3, p.12)

As the cited GP said, helping patients who are dying is for him one of the most rewarding elements of his daily work and is a key element in work satisfaction. On the other hand, some GPs sometimes found caring for dying patients to be emotionally heavy and burdensome, but this did not outweigh the sense of it also being rewarding.

(3) The responsibility of a GP was felt strongly by the following GP, especially when a patient died.

"As a GP, you feel responsible, so when someone dies suddenly you think, what was it? And: could I have prevented it?" (GP 6, p.7)

"A judge can judge a GP on this when it goes wrong." (GP 6, p.6)

(4) Many of the GPs felt they held a privileged position as a doctor, because they were able to become involved in very personal situations. This GP was amazed that he should share in such an intimate situation as the moment of dying.

"Then you can just sit next to it and watch it. How someone does it, shows it or indicates it. Then I think that is something just given to us, freely and at no cost." (GP 3, p.9)

Another aspect of the privileged position of a GP was learning from patients. One of the GPs said that her experiences with dying patients changed her own understanding of death and dying. She was able to test her theoretical framework by practical experience, which her brothers who did not work in health care were unable to do.

"And if you don't see it up close and personal, if you don't see people who are ill or about to die or dying or who have just died, then you're never confronted with how that all works. And then maybe you hold on to your own theories about that. My brothers, who don't work in health care, they're not confronted with these things and so they have a whole theoretical framework on what they think about the meaning of life. But it's never been tested in practice, the whole meaning of living and dying." (GP 8, p.4)

Several of the GPs who were also SCEN doctors indicated that they had changed their opinion about euthanasia as an option for themselves. The reasons they gave for this change were that they now realized that euthanasia is a failure to accept responsibility for one's own body; euthanasia means bothering somebody else; death is connected with life which one must accept and manage on one's own; euthanasia is not courageous; and lastly, euthanasia precludes making a natural farewell.

(5) When the GPs talked about their patients who had died they mentioned conditioning factors in contemporary society, in particular the out-of-hours service and the introduction of medical technology at home. The introduction of the out-of-hours service had a double impact. On the one hand it obliged GPs to care for dying patients who were not their own, and sometimes this required them to establish the cause of death in the case of an unknown patient and to complete the necessary papers for it. Some GPs said this was not part of their role, for in this situation the technical-medical responsibilities were detached from the person of the patient.

"It's bizarre. You get called out to completely unfamiliar people, you do your trick, you leave and in the car you make jokes with the driver." (GP 10, p.11)

On the other hand, the out-of-hours service obliged the GP to leave the care of his own patients who were dying to the GP who was on duty, and this he did not like doing. By way of a solution, many GPs suggested providing the carers of the dying patient with the GP's private telephone number.

*"I do always give people my phone number, but I'm not always home in the weekend. I would much rather go there on a Saturday night for a while and have him die a few hours later, than receive a note from the out-of-hours service on Monday morning. Because that way, it's not finished for me, I do like to finish the process." (GP 13, p.6)*.

The introduction of medical technology at home was seen as an obstacle for intimacy.

"What's become an important item for me in recent years, is that I notice that due to all kinds of technical circumstances and organizations involved, the intimacy of dying within a family is something you almost have to do battle over. That really bothers me." (GP 12, p.4)

### Personal values and experiences

The second group of family resemblance was to be found in personal values and experiences. The GPs expressed their personal values and experiences in talking about (1) the reasons why they remembered the death of a patient; (2) the GP as a human being with emotions and responsibility for his own care; (3) GPs' views on death in general and in particular their own; (4) moving the boundaries of their own values; and (5) the sense of responsibility the GPs showed in caring for their dying patients.

(1) The reasons why certain patients were remembered were all of a personal nature. The first reason was the person of the patient or the doctor. The GP felt respect or admiration for the patient because they showed a zest for living, a fighting spirit or authenticity. Sometimes the doctor identified with the patient, especially when the patient was his own age, or that of his parents or his children. The second reason for remembering was the quality of the personal relationship between the doctor and the patient, similar to that between a grandfather and grandchild, or characterised by a strong commitment or sympathy. The third reason was the memorable circumstances of the death. A death was regarded as positive when it was described as 'beautiful' or 'good', with time for saying farewell, when the communication between doctor and patient was good, and when life was rounded off well. A death was regarded as negative when the patient had been treated too long, had been referred unnecessarily to hospital or had died during the GP's holiday. It was also considered negative by the GP when a patient resisted dying or presumed they could choose the timing of their own death. The fourth reason for remembering a patient was when strong emotions were involved. These emotions included feelings of guilt or were associated with nightmares after the death of a patient. Another example of strong emotions was when the GP and the patient said farewell, just before euthanasia was performed, sometimes with a kiss or flowers for the doctor, as a token of thanks.

"If you're asking which euthanasias I still remember, well, it's the old ladies who say, give me a kiss, those I treasure the most, yes, give me a kiss." (GP 1, p.8)

(2) As a second topic involving personal values, the GPs discussed their own emotions and the care of themselves. The GP must deal with his own emotions as well as being aware of those of his patients and their carers. It seemed that younger GPs felt less emotional involvement than older GPs. They seemed to be focused more on the circumstances than on the personality of the patient. Older GPs, on the other hand, emphasized more the way in which the personality of the patient had impressed them.

The GPs observed two aspects of caring for a dying patient: it bestows energy and absorbs energy. The death of a patient is more overwhelming when the GP identifies with him, for instance when the patient is the same age as the GPs children.

"The age of such a girl is certainly a factor. I myself have a sixteen year-old daughter. That is always a factor and then it is written on the casket, bye sweetheart, and I often say bye sweetheart to my daughter. Well, my heart just fits to burst when I read that." (GP 15, p.16)

One GP said that the death of a patient could sadden him; another that he had once been deeply distressed by the death of a patient. A GP said that he immediately remembered the death of a child when he met the child's mother again after a lapse of 18 years.

Sometimes there is a conflict of roles, as in the case of a GP who wept at the funeral of a patient and then realized this was not expected of him.

"I know it's inappropriate, but it got away from me. But alright, I'll try to put the normal doctor's face back on. People thought that was more appropriate as well." (GP 17, p.10)

When strong emotions are involved self-care becomes essential. For one GP this meant reflecting on his own limits as a professional. This happened when he felt abused by a patient or sensed he was being played off against a member of the patient's family. Many GPs spoke of stressful situations in which their personal involvement had become too close.

How did GPs pick up their work again after the death of a patient? One of the GPs took a day off immediately after the death, another went out for a walk while a colleague took over his surgery hours, and a third did some menial tasks that did not require much thinking.

What I do then is potter around in the practice a little bit, just clearing some books or something, kill some time, that's really what it comes down to." (GP 10, p.20)

Sometimes GPs had difficulties sleeping at night. Some decided to reduce their working hours temporarily or even permanently in order to have more time for coping with their emotions. GPs could also share their emotions at home and found that sometimes a few words with their partner were sufficient to bring relief.

(3) Another personal topic that was discussed in the interviews was the GPs' views on the meaning of suffering and death in general and of their own death in particular.

One GP said that confrontation with dying patients had changed her view on the tasks one is given to fulfil in life.

"[Through guiding dying patients] I have actually changed my view on the tasks people get in life, whether life has meaning or not." (GP 8, p.4)

One GP was confronted with his own death while undergoing heart surgery and this had changed his perspective on life. It was the book of Psalms in the Bible that had given him a firm spiritual footing. He therefore saw it as his job to talk about spirituality with patients who knew they were going to die. He used religious or secular poetry or art to reach their hidden depths and in this way to contribute to their healing.

"You come to realize that for other people, certain things may matter in life that can help them get through those moments. I try to point this out to them: what matters to you now? Then we have to examine that more closely, those moments of essence." (GP 7, p.14)

Another GP interpreted the dying process as a source of strength hidden in a person, almost something holy, similar to the experience of a woman in the pangs of labour.

"What I sometimes see in dying, I would almost call it some primordial strength that shows itself in people who are dying. To me, it's somewhat comparable to women giving birth." (GP 3, p.8)

"It has to do with resignation, all at once getting a kind of wisdom on what life and dying is. And how to do it or why you could be at peace with that. [...] Those people are suddenly much stronger than all that surrounds them. They are above matter, as it were. [...] In our bodies there are hidden corners yet, that contain something that matters. [...] It gives me the feeling that yes, something in this dying is good." (GP 3, p.9)

The experiences one GP had accumulated as a doctor provided him with the resources for formulating his view on the meaning of suffering and death.

"Entering this world has suffering in it and leaving it also contains a bit of suffering. It also goes with humiliation, yes that too." (GP 1, p.4)

A doctor's experiences become the stimulus for thinking about his own death. Many GPs actually talked about their own death without being asked about it. Some had suffered a severe illness and had been close to death themselves. This had changed their attitude to dying patients and their own view of death. They described their ideal death as being not sudden but proceeded by a process that was not too long, as taking place in the intimate environment of home and with time for saying farewell properly. The purpose of a good farewell was to strengthen the ability of the relatives to cope with grief. One GP thought that suffering was an integral part of illness and recognised this could be true for her personally if illness struck.

Many GPs mentioned the mutual connection between the death of a patient and their own death. On the one hand, the GP is able to choose his own way of dying from the many ways in which he has seen his patients die and say farewell.

"I thought to myself, so it can be like this too, I would want it like this." (GP 2, p.5)

On the other hand, one GP projected his own ideas of a good death on his patients.

"I think that people should die well, if at all possible. If it is, then it is my job to make sure that happens. Those are probably the same elements I would want for my patients." (GP 3, p.10)

(4) In addition to the change in a GP's values over time, there was also a moving of the boundaries of values depending on the patient whom the doctor cared for. Some GPs described a tension in relationships with patients in whose case they changed their values at that moment. It seemed that GPs' values were not fixed, but dependent on the particular patient who was involved. One GP was against euthanasia, but agreed in favour of it for a patient who was determined to get it one way or another.

"I'm not very enthusiastic about euthanasia, you probably gathered that, but there are situations imaginable in which it is inescapable and in such cases, you really want your hands free." (GP 7, p.13)

Another GP talked about a very close and meaningful relation with a patient who asked for euthanasia. On principal, the GP was a supporter of euthanasia, but with this patient she hesitated.

"...and then I told [the SCEN-doctor] straight-up, that my bond with that man was far too strong for me, and that I really wanted it for him, but that it was extremely hard for me." (GP 2, p.8)

There seemed to be a mutual influence between the values and experiences of the doctor on the one hand and of the patient on the other. Obviously, the decisive influence of the GP's personal or professional values is determined by the actual relationship he has with the unique patient he is caring for at the time. The wishes of the patient may or may not fit in with the doctor's values. If they did not, the GP sometimes deviated from his value for the sake of one particular patient.

(5) Finally, several GPs formulated, implicitly or explicitly, their mission in caring for dying patients. They talked passionately about their goals, such as self-care for the GP, a view of life as the inspiration for their work, the precept 'Do good' as a motto both for life and work, ensuring intimacy for a patient on their deathbed, the uniqueness of each person's path in life, and the importance of a good farewell before dying.

### A good death or deathbed

The third family of thematic resemblances dealt with descriptions of a good death or a good process of dying. The GPs eventually wanted to clarify what they meant by a good death or deathbed. According to them, a good death has several characteristics: one is the autonomy of the patient. The following GP described the importance of the right use of autonomy being expressed by a good farewell and a willingness to depend on others and submit to them at a certain point on the way to death.

"You may plan the farewell beautifully, but there should also be a moment in which you really surrender yourself. That you are really dependent on the surroundings. However hard that may be sometimes." (GP 9, p.5)

One GP mentioned several times the importance of a death in harmony with the person of the patient, including the moment of dying and the way of dying. Other GPs mentioned the importance of the patient's philosophy of life. A religious world view can be very significant in the dying process as it may bring strength, peace, trust and comfort to the dying patient as well as to their loved ones. A good death was also associated with a healthy balance between professional distance and personal nearness to the patient. Many stories told by the GPs described the difficulty of attaining this balance. One GP developed a deep relationship with an elderly patient whom she visited socially every week. She talked about her hesitation as to whether this was sound in terms of the relationship between doctor and patient.

"I found it hard to assess to what extent it is normal to go there every week as a GP, and drink coffee and eat cake." (GP 2, p.10)

Altogether, the GPs who were interviewed described the ideal death of a patient. The patient should be aware he is dying and accept that fact; ideally his view of life could help him to do this. The patient wants to die naturally, at home, without euthanasia, and without a lot of technological assistance. The patient should be in control until just before the moment of death and then he should submit. Lastly, the environment should be helpful. In addition to the patient being in control of the situation, the GP himself should also be able to practise his role as backstage director and guide of the patient.

## Discussion

This study resulted in a didactic doctor-patient model which focuses on the GP and what important aspects need attention in dealing with a patient's death. The model clarifies the uniqueness and reciprocity of the doctor-patient relationship in the situation of the patient's death. The GP's professional and personal values and experiences are unique and interact with the patient's values. The development of this unique relationship between doctor and patient seems to be decisive for the care given to the patient and may change over the course of time.

This has consequences for medical education. Because the actions of the doctor are being driven by professional and personal values and experiences, especially in caring for dying patients, GPs must be aware of their own values and past experiences of death and dying [26]. The way in which GPs have handled their own grief and mourning at times of loss is certainly a major factor in their own subsequent ability to accept and mourn the loss of a patient and to help patients and families in dealing with their grief. The model that is the outcome of this study supports reflection as an integral part of GP training [27]. As our study reveals that values are not fixed but may change over time, reflection may also be used as a framework for future learning done in the process of practice [28].

The main themes are to be understood in the Wittgensteinian sense of family resemblance: the main themes we identified (professional values, personal values, and good death) are unifying terms that do not want to suggest that they are connected by one essential feature. Rather, the main themes compile a multitude of topics that are connected by "a complicated network of similarities, overlapping and criss-crossing" [29]. In addition, the three main themes themselves also have family resemblances: In the heart of the model are three themes that connect statements about professional values and experiences, personal values and experiences and descriptions of how GPs understand a good death or a good dying process. These groups of statements overlap. For instance, opinions about euthanasia could arise from professional as well as from personal values. If, furthermore, the opinion is backed by a favourable attitude towards euthanasia as a desirable way of dying, it also belongs to the category 'a good death'.

### Comparison with other models

Our model covers both values and experiences (emotions) as most other models do. In addition, it also shows the mutual influence between the values of the doctor and those of the patient. However, our model does not explicitly cover the spiritual dimension as Leget's model does with the themes of 'life balance and guilt', and 'death and the afterlife'. These themes were mainly discussed in our interviews when they arose in the context of personal experiences with death. It is likely that these topics were not considered to belong to the GP's domain or that the GPs did not feel either free or able to discuss them. In a Scottish interview study most GPs said they had a role in providing spiritual care, but hesitated to raise spiritual issues with the patient, either on account of a lack of time, or a feeling that they should wait for a cue, or an awareness of their being unprepared or unskilled [30].

One of the striking results of our study is that it seemed that several of the GPs who took part had changed their views on dying as a result of their cumulative experiences in the course of their career. Some GPs, who were also SCEN-doctors, said they did not want euthanasia for themselves. The factors that might have influenced their opinion on the subject were increasing age, wider professional experience or developments in palliative care. Elderly persons are closer to their own death and may have a greater acceptance of suffering. It is also possible that human tolerance of suffering may increase over time. Another explanation is that experienced GPs have seen more people die well and they think that their patients deserve a good death too. A greater ability for reflection on the part of experienced GPs may enable them to distance themselves from their patient's suffering. It seems that experienced GPs accept suffering better because suffering belongs to dying. On the other hand, it is also possible that where the relationship between doctor and patient has been longstanding the GP may not accept that the patient should suffer because he or she has become too dear to him. As a final explanation, the improvement in palliative care in regard to sedation and pain management over the course of the GP's career might have changed his views about euthanasia.

### Strengths and limitations of this study

Although we wanted to include a wide diversity of opinions, our GPs were from only one part of the Netherlands, and from one ethnicity (white and western). Since we found that younger GPs were more focused on the circumstances of a death and older ones on the personality of the patient, the selection of more senior participants might have influenced the results of our study.

Yet, it is unsure whether these limitations had a large impact on the topics discussed. The variety of topics discussed in our interviews seems very wide. Of the 23 factors involving risk for a physician's feelings that can influence patient care, as found by Meier [17], all but three can be traced in our study. Those not discussed were psychiatric illness in the case of the doctor, physicians' disagreements with colleagues over patient management, and the question of circumstances in which the patient is well known.

Also, when we compare the 43 themes from our interviews (Table 3) with the broad topics found in the literature, we find that the consideration of only the cultural context and education was missing among our themes. Although two practices were selected because a large number of their patients come from minority groups, the different cultural or religious contexts of patients were not discussed. The reason may be found in the degree of adaptation to western culture by the minority groups, which would account for patients keeping to themselves their most private feelings with regard to tradition, culture and religion, or the reason may be language barriers between doctor and patient. The GPs did not talk about educational factors such as preparation for dealing with dying patients either. Evidently, they did not feel that their education had been incomplete or that the matter was of great importance.

## Conclusions

As the GPs' opinions on death and dying influence the kind of patient care they give, it is important to teach GPs and GP-trainees to reflect on these experiences. Values are not fixed but appear to change over time and to depend on the unique relationship between doctor and patient. Our doctor-patient model might be a tool for clarifying the complex situation of the GP. In that way, our study, as part of narrative-based medicine, might be used as a resource for health care education.

## Competing interests

The authors declare that they have no competing interests.

## Authors' contributions

AAHV was the principal investigator and coordinated the inclusion of the participants of the study, carried out the individual interviews and drafted the manuscript. All the authors participated in the conception and design of the study, in the analysis and interpretation of the data, and in revising the manuscript. All of them read and approved the final manuscript.

## Pre-publication history

The pre-publication history for this paper can be accessed here:

http://www.biomedcentral.com/1471-2296/12/8/prepub

## Supplementary Material

Additional file 1 **Questionnaire**. GPs'questionnaire on personal and practice characteristics. Click here for file

Additional file 2 **The doctor-patient model**. The doctor-patient model with interactions of values and experiences of the doctor when confronted with the death of a patient. Click here for file

Additional file 3 **Characteristics of GPs**. Personal and practice characteristics of GPs participating in interviews about the death of a patient. Click here for file
