# Supplementary material for: The death of a patient: a model for reflection in GP training
Source: BMC Fam Pract. 2011 Mar 3;12:8. doi: 10.1186/1471-2296-12-8 (PMC3061910; doi:10.1186/1471-2296-12-8)
Supplement: Additional file 1 — Questionnaire. GPs'questionnaire on personal and practice characteristics. [file 1471-2296-12-8-S1.DOC]

## Appendix 1 – Questionnaire

| Name:  Date: |  |  |
| --- | --- | --- |
| 1. Sex |  | 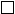 Male  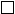 Female |
| 2. Age |  | years |
| 3. How many years of professional experience do you have? |  | years |
| 4. What percentage of your time do you work? |  | % |
| 5. How many patients does/did your practice have? |  | patients |
| 6. What kind of practice do you have? |  | 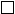 urban  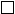 rural  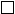 urbanized rural |
| 7. Do you practice as a physician |  | 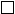 alone in a building   - together with other physicians in a building |
| 8. How do live in your personal situation? |  | 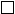 alone  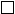 with others |
| 9. Do you call yourself religious? |  | 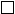 yes  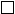 no |
| If not, do you have an explicit world view? |  | 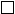 yes  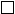 no |
