# Supplementary material for: The death of a patient: a model for reflection in GP training
Source: BMC Fam Pract. 2011 Mar 3;12:8. doi: 10.1186/1471-2296-12-8 (PMC3061910; doi:10.1186/1471-2296-12-8)
Supplement: Additional file 2 — The doctor-patient model. The doctor-patient model with interactions of values and experiences of the doctor when confronted with the death of a patient. [file 1471-2296-12-8-S2.PPT]

## Slide 1
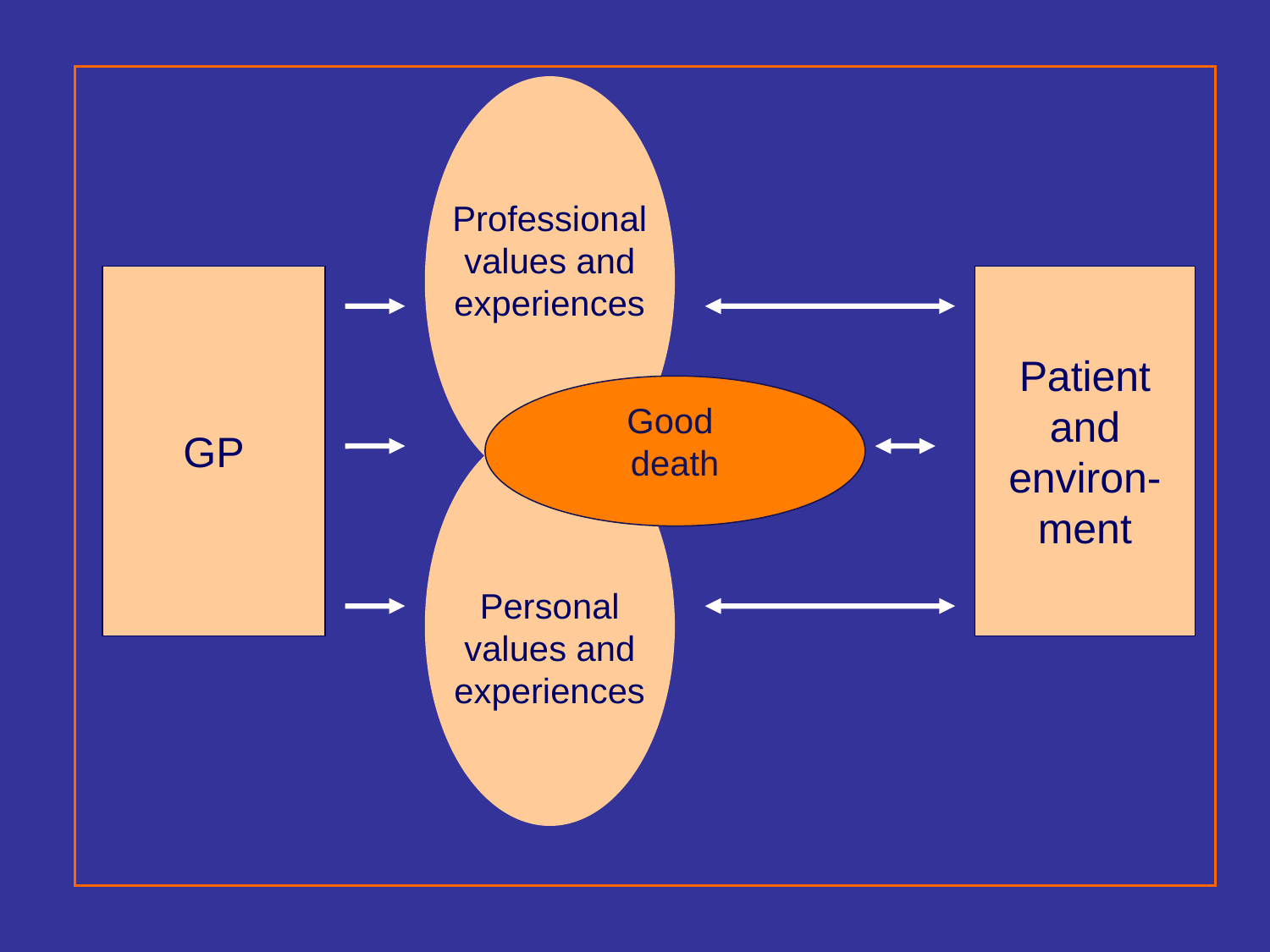

Professional
values and
experiences
GP
Patient
and
environ-
ment
Good
death
Personal
values and
experiences
