# Supplementary material for: The death of a patient: a model for reflection in GP training
Source: BMC Fam Pract. 2011 Mar 3;12:8. doi: 10.1186/1471-2296-12-8 (PMC3061910; doi:10.1186/1471-2296-12-8)
Supplement: Additional file 3 — Characteristics of GPs. Personal and practice characteristics of GPs participating in interviews about the death of a patient. [file 1471-2296-12-8-S3.DOC]

## Appendix 2 - Personal and practice characteristics of participating GPs

| GP^1^ | Date of interview  in 2007^2^ | Sex, age category ^3^ (yrs) | Experience (yrs) | Employment  (%) | Practice  size (number of patients) | Type of practice^4^ | Religious? If not: explicit view of life? |
| --- | --- | --- | --- | --- | --- | --- | --- |
| 1 | 8 May | M, 50-60 | 27 | 70 | 1600 | Urban | No; no |
| 2 | 24 Aug | F, 50-60 | 26 | Not filled in | 1500 | Urbaniz-ed rural | No; no |
| 3 | 17 Sept | M, 50-60 | 30 | 100 | 2600 | Urban | No; no |
| 4 | 28 Sept | M, 60-65 | 28 | 0,5 | 2000 | Rural | No; yes |
| 5 | 9 Oct | F, 40-50 | 15 | 50 | 1300 | Urbanized rural | Yes |
| 6 | 26 Oct | M, 40-50 | 8 | 100 | 2350 | Urban | Yes |
| 7 | 15 Nov | M, 50-60 | 31 | 70 | 1800 | Urban | Yes |
| 8 | 7 Dec | F, 30-40 | 7 | 70 | 2500 | Urbanized rural | Don’t know; no |
| 9 | 10 Dec | M, 50-60 | 27 | 80 | 1600 | Urban | Yes |
| 10 | 11 Oct | M, 50-60 | 30 | 80 | 2600 | Urban | No; yes |
| 11 | 11 Oct | M, 60-70 | 33 | 20 | 3750 | Urban | No; no |
| 12 | 11 Oct | M, 60-70 | 34 | 100 | 5100  3 GPs | Urban | No; no |
| 13 | 11 Oct | M, 50-60 | 22 | 100 | 2200 | Rural | No; no |
| 14 | 11 Oct | M, 60-70 | 40 | 0 | 8000  more GPs | Urban | Yes |
| 15 | 11 Oct | M, 50-60 | 25 | 100 | 2350 | Urbanized rural | Yes |
| 16 | 11 Oct | M, 50-60 | 32 | 100 | 3100 | Urban | No; no |
| 17 | 11 Oct | M, 50-60 | 27 | 100 | 2750 | Urban | No; yes |
| 18 | 11 Oct | M, 50-60 | 21 | 80 | 5600  3 GPs | Urbanized rural | Yes |

^1^ GPs 2, 3, 4, 5 and 10-18 are also SCEN-physicians

^2^ GP 1 - 9 were interviewed individually, GPs 10-18 participated in a group interview (focus group)

^3^ To protect anonymity only the age category is mentioned.

^4^ Urbanized rural means a practice in a rural community in transition to an urban environment
